# Curcumin Effectively Rescued Parkinson's Disease-Like Phenotypes in a Novel *Drosophila melanogaster* Model with dUCH Knockdown

**DOI:** 10.1155/2018/2038267

**Published:** 2018-07-03

**Authors:** Thi Thanh Nguyen, My Dung Vuu, Man Anh Huynh, Masamitsu Yamaguchi, Linh Thuoc Tran, Thi Phuong Thao Dang

**Affiliations:** ^1^Department of Molecular and Environmental Biotechnology, Faculty of Biology and Biotechnology, University of Science, Vietnam National University-Ho Chi Minh City, Ho Chi Minh City 700000, Vietnam; ^2^Department of Applied Biology, Kyoto Institute of Technology, Kyoto 606-8585, Japan; ^3^The Center for Advanced Insect Research, Kyoto Institute of Technology, Kyoto 606-8585, Japan; ^4^Laboratory of Molecular Biotechnology, University of Science, Vietnam National University-Ho Chi Minh City, Ho Chi Minh City 700000, Vietnam

## Abstract

The relationship between oxidative stress and neurodegenerative diseases has been extensively examined, and antioxidants are considered to be a promising approach for decelerating disease progression. Parkinson's disease (PD) is a common neurodegenerative disorder and affects 1% of the population over 60 years of age. A complex combination of genetic and environmental factors contributes to the pathogenesis of PD. However, since the onset mechanisms of PD have not yet been elucidated in detail, difficulties are associated with developing effective treatments. Curcumin has been reported to have neuroprotective properties in PD models induced by neurotoxins or genetic factors such as *α-synuclein*, *PINK1*, *DJ-1*, and *LRRK2*. In the present study, we investigated the effects of curcumin in a novel *Drosophila* model of PD with knockdown of dUCH, a homolog of human UCH-L1. We found that dopaminergic neuron-specific knockdown of dUCH caused impaired movement and the loss of dopaminergic neurons. Furthermore, the knockdown of dUCH induced oxidative stress while curcumin decreased the ROS level induced by this knockdown. In addition, dUCH knockdown flies treated with curcumin had improved locomotive abilities and less severe neurodegeneration. Taken together, with studies on other PD models, these results strongly suggest that treatments with curcumin are an appropriate therapy for PD related to oxidative stress.

## 1. Introduction

The imbalance between reactive oxygen species (ROS) generation and cellular antioxidant activity leading to oxidative stress is known to be a major cause of neurodegenerative diseases such as Alzheimer's disease (AD) and Parkinson's disease (PD) [1]. Therefore, antioxidants are regarded as a potential treatment for neurodegenerative disorders including PD [2, 3]. PD, also known as shaking palsy, was initially described by Dr. James Parkinson in 1817 [4]. PD affects 1% of the population aged older than 60 years and is considered to be the second most common neurodegenerative disorder after AD. PD is characterized by progressive impairments in locomotive ability such as tremor, rigidity, and bradykinesia. These symptoms are attributed to the loss of dopaminergic neurons (DA neurons) in the substantia nigra and the formation of Lewy bodies in the brain [5–7]. The complex interaction between environmental and genetic factors results in PD; however, the interactions between these factors have not yet been elucidated in detail. Previous studies implicated mitochondrial dysfunction, oxidative stress, altered proteolysis, and inflammation, in the pathogenesis of PD [8–10].

Many genes and their variants, such as *α*-*synuclein*, *PINK1*, *DJ-1*, *UCH-L1*, and *LRRK2*, have been shown to contribute to the pathogenesis of PD [8]. Ubiquitin carboxyl-terminal hydrolase L1 (UCH-L1) belongs to a family of deubiquitinating enzymes (DUBs), which plays important roles in the ubiquitin-proteasome system (UPS). UCH-L1 is present predominantly in the brain and accounts for 1–5% of all neuronal proteins [11]. In 1998, a missense mutation in UCH-L1 (I93M) was initially identified in a German family with PD [12]. In 2013, another missense mutation in UCH-L1 (E7A) was found in three siblings with early-onset progressive neurodegenerative syndrome including optic atrophy, spasticity, and ataxia [13]. In contrast, another variant of UCH-L1 (S18Y) was discovered as a factor in the risk reduction of PD [14]. Other studies reported that UCH-L1 was related to the abnormal accumulation and aggregation of *α*-synuclein, which leads to the formation of Lewy bodies [15].

There is currently no potent therapy to cure PD, and medication merely supports the control of symptoms. Therefore, many cellular and animal models of PD have been developed with the purpose of drug discovery [16–19], and these models may be classified into two major groups: toxin and genetic models [20, 21]. Previous studies using *in vitro* and *in vivo* models showed that curcumin exerted protective effects on PD [22–25]. In the dopaminergic cell lines MES23.5 and SH-SY5Y, curcumin treatments attenuated the neurotoxicity of 6-OHDA and improved cell viability by inhibiting increases in ROS levels, mitochondrial protection, and reducing p53-mediated apoptosis [22, 23]. A recent study also reported that curcumin protected against mitochondrial dysfunction and cell death in PINK1 knockdown SH-SY5Y cells [24]. Moreover, curcumin had the potential not only to increase lifespan and locomotor abilities but also to decrease oxidative stress and DA neuron degeneration in rotenone-induced and human *α*-synuclein-expressing *Drosophila* models of PD [26, 27]. PD-like phenotypes in a fly model induced by the combination of a H_2_O_2_ treatment and a *LRRK2* mutation were suppressed by curcumin through decreases in oxidized protein levels and LRRK2 kinase activity [28]. A previous study using a 6-OHDA rat model showed that a curcumin treatment protected the number of DA neurons in the substantia nigra as well as dopamine levels [29]. Additionally, curcumin attenuated MPTP-induced neurotoxicity and locomotor defects in a PD mouse model by protecting against oxidative stress and suppressing *α*-synuclein aggregation [30]. In the DJ-1 knockout rat, liposomal-formulated curcumin targeting histone deacetylase ameliorated motor impairments and prevented apoptosis [31].

*Drosophila* is a suitable model for studying human neurodegenerative diseases [32, 33]. In *Drosophila*, ubiquitin carboxyl-terminal hydrolase (dUCH) encoded by the *CG4265* gene is a homolog of human UCH-L1. Therefore, we used a *Drosophila* model to study the link between dUCH knockdown, oxidative stress, and PD. Furthermore, we also evaluated the effects of curcumin on PD-like phenotypes caused by DA neuron-specific knockdown of dUCH in this model.

## 2. Materials and Methods

### 2.1. Fly Stocks and Food Preparation

Fly stocks were maintained on standard food containing 1% agar, 5% sucrose, 5% dry yeast, and 3% powdered milk at 25°C. Curcumin (purity ≥ 65%, C1386, Sigma-Aldrich, USA) was dissolved in DMSO and then added to standard food at a final concentration of 0.037% which is equivalent to 1 mM of curcumin. Curcumin-containing medium was kept in the dark to avoid its photooxidation during experimental procedures. The RNAi line carrying *UAS-dUCH.IR* (v26468, Vienna Drosophila Resource Center (VDRC)) was used to knock down dUCH, and wild-type Canton-S (Bloomington Drosophila Stock Center (BDSC)) was used to create control flies. The UAS-RNAi line was driven by *TH-GAL4 (ple-GAL4)* (8848, Bloomington Drosophila Stock Center) or *GMR-GAL4* [34].

### 2.2. *In Vivo* ROS Detection

The ROS assay was performed as described previously [35] with some modifications. Eye imaginal discs from the third instar larvae or brains from adult flies were dissected in PBS and then fixed in 1% paraformaldehyde for 5 minutes. After being washed by PBS, these tissues were incubated with 10 *μ*M CM-H2DCFDA (C6827, Life Technologies, USA) for 15 minutes. The samples were washed with PBS three times and then mounted in Vectashield Mounting Medium (Vector Laboratories, Japan). The samples were inspected under a confocal laser scanning microscope (Olympus Fluoview FV10i) or an Olympus BX41 microscope.

### 2.3. Feeding Assay

In this assay, Coomassie Brilliant Blue dye was added to the fly medium in order to quantify food intake. The early third instar larvae were collected and washed with PBS. These larvae were then transferred into food containing Coomassie and allowed to eat for 30 minutes. The larvae were washed with 70% ethanol and water and homogenized in 70% ethanol. The homogenates were then centrifuged at 9279 ×g for 10 minutes. The quantities of Coomassie in the supernatants were measured at OD595. This procedure was repeated four times with 50 larvae each time.

### 2.4. Crawling Assay

The crawling assay was performed as described previously [36] with some modifications. Forty male larvae in the early third instar stage were collected randomly and washed with PBS to discard food traces. After that, larvae were transferred to agar plates containing 2% agar with a density of 2–4 larvae per plate. The movement of larvae was recorded by a digital camera for 60 seconds. The recorded videos were then converted into AVI files using a MOV to AVI converter (Pazera Jacek, Poland) and analyzed by ImageJ (NIH, USA) with the wrMTrck plugin (developed by Dr. Jesper Søndergaard Pedersen) to track larval movement and draw motion paths.

### 2.5. Climbing Assay

The climbing assay was performed as described previously [37]. Thirty newly eclosed adult male flies were collected and transferred to cylindrical tubes with a height of 15 cm and a diameter of 2 cm. The tubes were tapped to collect the flies in the bottom, and the movements of flies were recorded for a duration of 30 seconds. These procedures were repeated five times and recorded by a digital camera. In all climbing experiments, the height to which each fly climbed was scored as follows: 0 (less than 2 cm), 1 (between 2 and 4 cm), 2 (between 4 and 6 cm), 3 (between 6 and 8 cm), 4 (between 8 and 10 cm), and 5 (more than 10 cm). The climbing assay was performed every five days until all flies lost their locomotor abilities.

### 2.6. Quantification of Dopaminergic Neurons by Immunostaining

Brains from the third instar larvae or adult flies were dissected in PBS and fixed with 4% paraformaldehyde for 20 minutes. After being washed by PBS containing 0.3% Triton X-100, samples were blocked by PBS containing 0.15% Triton X-100 and 10% normal goat serum at 25°C for 30 minutes. Tissues were then incubated with a diluted primary antibody, rabbit anti-tyrosine hydroxylase (anti-TH) (1 : 250, Millipore), in PBS containing 0.15% Triton X-100 and 10% normal goat serum at 4°C for 36 hours. After being washed, brains were incubated with a secondary antibody conjugated with Alexa 488 (1 : 500, Invitrogen) at 25°C for 2 hours. Tissues were then washed and mounted in Vectashield Mounting Medium (Vector Laboratories, Japan). Samples were observed under a confocal laser scanning microscope (Olympus Fluoview FV10i) or an ECLIPSE NI-U (Nikon).

### 2.7. Dopamine Quantification by High-Performance Liquid Chromatography (HPLC)

Thirty heads of adult flies were collected and homogenized in homogenization buffer containing 100 mM perchloric acid and 184 mM trichloroacetic acid. The homogenates were sonicated for 30 seconds on ice five times and then centrifuged. The HPLC was performed using an electrochemical detector (HPLC-ECD, Bioanalytical system) with running buffer containing 180 mM chloroacetic acid, 50 *μ*M EDTA2Na, 160 mM sodium hydroxide, 8.5% acetonitrile, and 1.7 mM sodium octane sulfate. The samples were separated by C18 columns (5 *μ*m, 100 mm × 1 mm) for phase 1 and C18 UniJet LC columns (3 *μ*m, 100 mm × 2 mm) for phase 2 (Bioanalytical system) at a flow rate of 0.5 ml/minute. Dopamine (H8502, Sigma-Aldrich) was used to establish the standard curve.

### 2.8. Data Analysis

GraphPad Prism 6 was used to perform statistical analyses. Tukey's test was used to analyze the results of ROS assays using eye imaginal discs, crawling assays, and the quantification of DA neurons, with error bars representing the standard deviation of data (SD). Data from the ROS assays using brains were analyzed with a one-way ANOVA, and error bars indicated SD. In feeding assays, *P* values were calculated using a one-way ANOVA and error bars indicated the standard errors of means (SEM). The results from climbing assays were analyzed using a two-way ANOVA, and error bars represented the SEM. In the results from the quantification of dopamine levels, error bars indicated the SD and interassay coefficients of variation (CV) were calculated.

## 3. Results

### 3.1. Knockdown of dUCH Induced Oxidative Stress while Curcumin Decreased ROS Level Induced by This Knockdown

**Figure 1:**
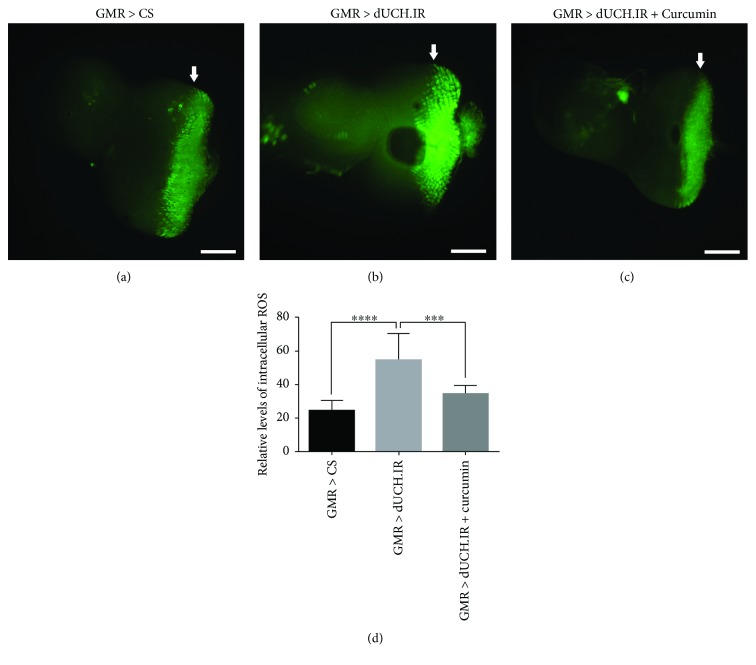
Curcumin decreased ROS levels induced by the knockdown of dUCH in eye imaginal discs. Detection of ROS levels in third instar larval eye imaginal discs by CM-H2DCFDA. ROS levels in the area posterior to the morphogenetic furrow in eye imaginal discs were higher in dUCH knockdown larvae (GMR > dUCH.IR) (b) than in control larvae (GMR > CS) (a). Eye imaginal discs of dUCH knockdown larvae treated with curcumin (GMR > dUCH.IR + curcumin) (c) showed decreased ROS levels in the area posterior to the morphogenetic furrow. White arrows indicate morphogenetic furrows, and scale bars indicate 100 *μ*m. (d) Quantified data of the intensities of the region posterior to the morphogenetic furrow in eye imaginal discs shown in (a–c) (Tukey's test; ^∗∗∗^*P* < 0.001 and ^∗∗∗∗^*P* < 0.0001; *n* = 11; error bars represent the standard deviation of data). GMR > CS (*GMR-GAL4/Y; +; +*) and GMR > dUCH.IR (*GMR-GAL4/Y; +; UAS-dUCH.IR/+*).

**Figure 2:**
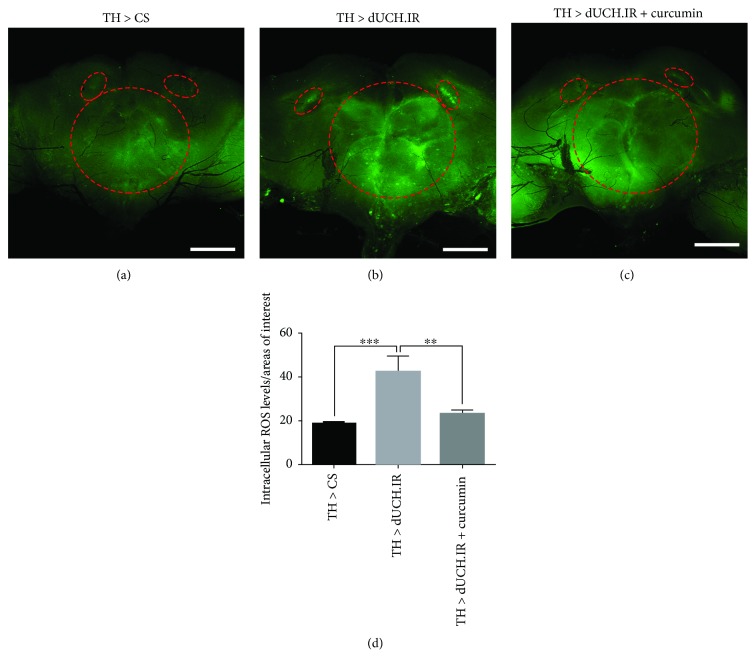
Curcumin decreased ROS levels induced by the knockdown of dUCH in adult brains. Detection of ROS levels in adult brains by CM-H2DCFDA. ROS levels were higher in fly brains with the knockdown of dUCH (TH > dUCH.IR) (b) than in control fly brains (TH > CS) (a). ROS levels also decreased in adult fly brains with the knockdown of dUCH treated with curcumin (TH > dUCH.IR + curcumin) (c). The red dashed circles enclose the regions of interest, and scale bars indicate 100 *μ*m. (d) Quantified data of the intensities of adult brains shown in (a–c) (one-way ANOVA; ^∗∗^*P* < 0.01 and ^∗∗∗^*P* < 0.001; *n* = 3; error bars represent the standard deviation of data). TH > CS (*w/Y; +; TH-GAL4/+*) and TH > dUCH.IR (*w/Y; +; TH-GAL4/UAS-dUCH.IR*).

With the aim of elucidating the relationship between the knockdown of dUCH and oxidative stress, we evaluated ROS levels in the eye imaginal discs and adult brains of dUCH knockdown flies. The results showed that ROS levels in eye imaginal discs were 2.3-fold higher in knockdown larvae carrying *GMR-GAL4/Y; +; UAS-dUCH.IR/+* than in control larvae carrying *GMR-GAL4/Y; +; +* (Figures 1(a), 1(b), and 1(d)). Similarly, we found an increase in ROS levels in flies with the specific knockdown of dUCH in DA neurons by using the *TH-GAL4* driver strain. ROS levels in the brain were 2.2-fold higher in knockdown flies (*w/Y; +; TH-GAL4/UAS-dUCH.IR*) than in control flies (*w/Y; +; TH-GAL4/+*) (Figures 2(a), 2(b), and 2(d)). These results suggest that the knockdown of dUCH increased ROS levels in *Drosophila*.

Previous studies demonstrated that curcumin has the potential to protect against cellular damage caused by oxidative stress [38]. We speculated that curcumin, which exhibits strong antioxidant activity, might decrease ROS levels induced by the knockdown of dUCH in eye imaginal discs and adult brains. As expected, curcumin decreased dUCH knockdown-induced ROS levels. ROS levels in eye imaginal discs were lower (0.6-fold) in curcumin-treated dUCH knockdown larvae (*GMR-GAL4/Y; +; UAS-dUCH.IR/+*) than in curcumin-untreated knockdown larvae (Figures 1(b), 1(c), and 1(d)). In adult brains, we also found that ROS levels were 0.5-fold lower in curcumin-treated dUCH knockdown flies (*w/Y; +; TH-GAL4/UAS-dUCH.IR*) than in untreated knockdown flies (Figures 2(b), 2(c), and 2(d)). Thus, these results clearly demonstrated that knockdown of dUCH induced ROS levels which might lead to oxidative stress.

### 3.2. Food Intake Ability of Larvae Was Not Influenced by the Curcumin Treatment

**Figure 3:**
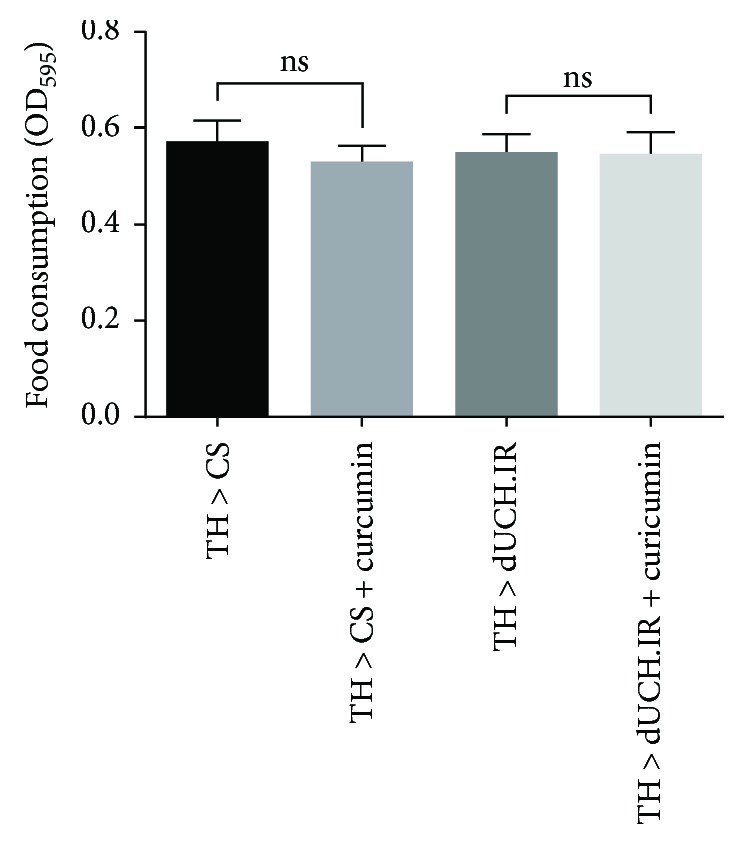
Curcumin did not affect larval food intake ability. dUCH knockdown larvae (TH > dUCH.IR) and control larvae (TH > CS) were treated with curcumin. The food intake ability of dUCH knockdown and control larvae did not change significantly when they were fed fly food containing curcumin (one-way ANOVA, ns: not significant *P* > 0.05, population size *N* = 50, and biological replication *n* = 4). Error bars indicate the standard error of the mean. TH > CS (*w/Y; +; TH-GAL4/+*) and TH > dUCH.IR (*w/Y; +; TH-GAL4/UAS-dUCH.IR*).

Curcumin is a powerful antioxidant and has already been studied as a promising therapeutic compound for PD in *Drosophila* models [26, 27]. Thus, we aimed to further investigate the effects of curcumin on the phenotypes of dUCH knockdown flies following the confirmation of a relationship between the knockdown of dUCH and oxidative stress. The taste of food has been suggested to have negative or positive effects on the amount of food consumed by animals, which may, in turn, have an impact on animal health. Therefore, we examined the influence of curcumin on larval food intake ability using a feeding assay. In this experiment, the *TH-GAL4* driver strain was crossed with flies carrying *UAS-dUCH.IR* in order to specifically knock down dUCH in DA neurons. No significant differences were observed in the food intake abilities of knockdown larvae (*w/Y; +; TH-GAL4/UAS-dUCH.IR*) and control larvae (*w/Y; +; TH-GAL4/+*) between the treatment and no treatment with curcumin (Figure 3). Therefore, curcumin had no effect on larval food intake ability. These results are consistent with previous findings on the effects of curcumin on larval feeding behavior [39].

### 3.3. Locomotor Defects Caused by the DA Neuron-Specific Knockdown of dUCH Were Improved by the Curcumin Treatment

**Figure 4:**
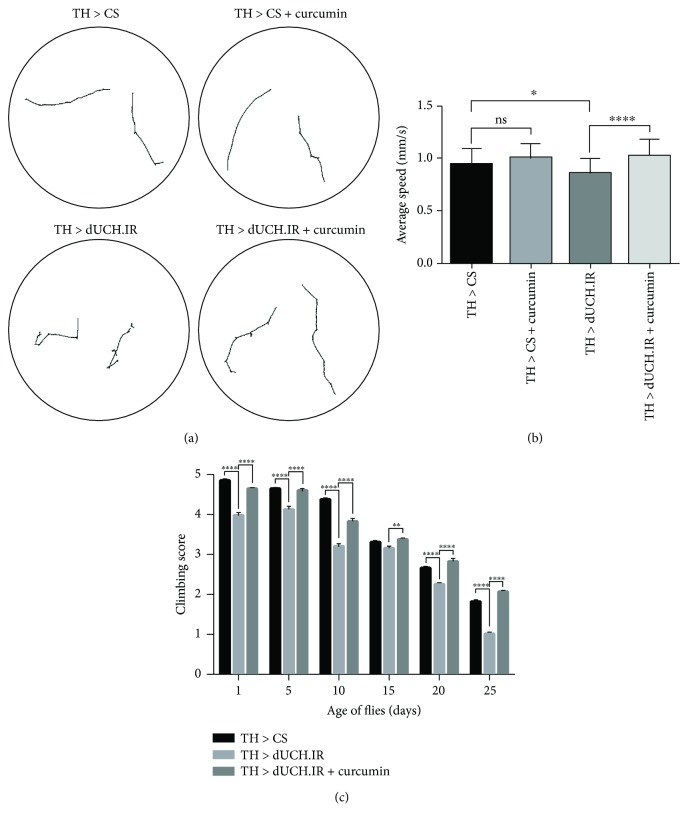
The curcumin treatment recovered locomotor defects from the dopaminergic neuron-specific knockdown of dUCH in larval and adult stages. (a) Images show the motion paths of control (TH > CS), control treated with curcumin (TH > CS + curcumin), dUCH knockdown (TH > dUCH.IR), and dUCH knockdown larvae treated with curcumin (TH > dUCH.IR + curcumin). (b) Quantified data of the crawling assay. Curcumin did not affect the crawling ability of control larvae. dUCH knockdown larvae crawled more slowly than control larvae, while dUCH knockdown larvae fed curcumin showed the recovery of crawling ability (Tukey's test; ^∗^*P* < 0.05 and ^∗∗∗∗^*P* < 0.0001; *n* = 40; error bars represent the standard deviation of data). (c) Climbing assay. The knockdown of dUCH in dopaminergic neurons caused a decline in climbing ability; however, dUCH knockdown flies treated with curcumin also showed the strong recovery of climbing ability (two-way ANOVA; ^∗∗^*P* < 0.01 and ^∗∗∗∗^*P* < 0.0001; population size *N* = 30 and technical replication *n* = 5; error bars indicate the standard error of the mean). TH > CS (*w/Y; +; TH-GAL4/+*) and TH > dUCH.IR (*w/Y; +; TH-GAL4/UAS-dUCH.IR*).

We then investigated the effects of curcumin on the locomotor abilities of dUCH knockdown flies by performing a crawling assay with the larval stage and a climbing assay with the adult stage. In the larval stage, the results obtained showed that dUCH knockdown larvae exhibited not only abnormal behaviors but also decreased crawling speeds. dUCH knockdown larvae (*w/Y; +; TH-GAL4/UAS-dUCH.IR*) crawled with tremor-like movement paths, and their crawling speed was 0.09 mm/s slower than that of control larvae (*w/Y; +; TH-GAL4/+*) (Figures 4(a) and 4(b)). dUCH knockdown larvae fed with curcumin exhibited recoveries in crawling behavior and speed, while the crawling ability of control larvae was not affected by curcumin. The crawling speed of curcumin-treated knockdown larvae was 0.16 mm/s faster than that of untreated knockdown larvae. These differences were significant (Tukey's test, *P* < 0.05, *n* = 40) (Figures 4(a) and 4(b)). Similar to the larval stage, the knockdown of dUCH led to a decrease in climbing ability in the adult stage. The results showed that dUCH knockdown flies started to exhibit less mobility on day 1 after eclosion than control flies and climbing ability continuously decreased from day 1 to day 25. However, dUCH knockdown flies fed curcumin exhibited improved locomotor ability, showing more mobility than curcumin-untreated knockdown flies at all-time points examined (Figure 4(c)). These results suggest that curcumin protects against the negative effects of the dUCH knockdown on locomotor abilities in the larval and adult stages.

### 3.4. Curcumin Treatment Reduced the Degeneration of Dopaminergic Neurons Caused by the Knockdown of dUCH in Larval and Adult Stages

**Figure 5:**
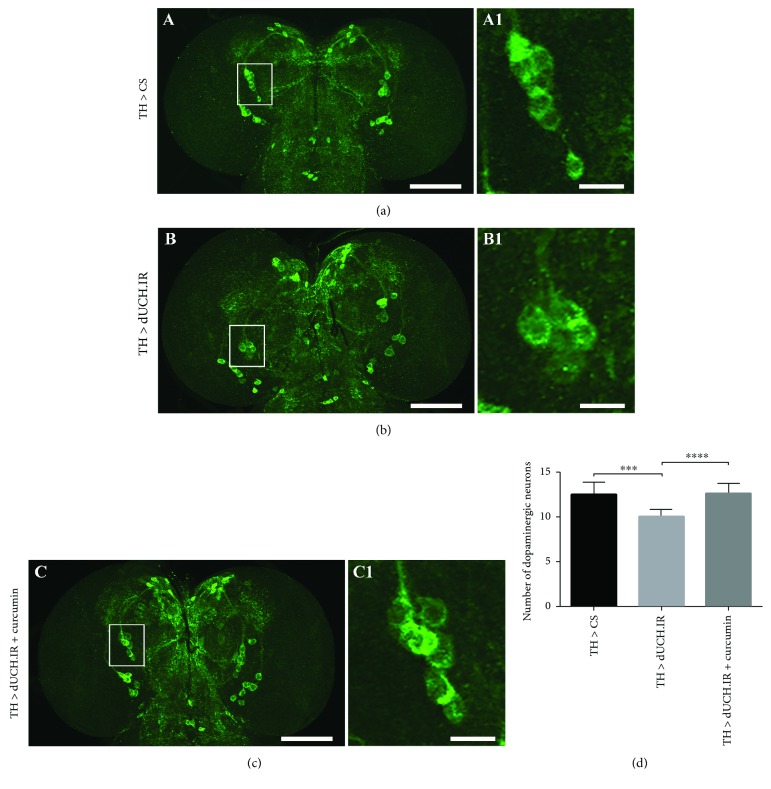
Curcumin reduces the degeneration of dopaminergic neurons in dUCH knockdown larvae. (a–c) Immunohistochemical images of larval brain lobes from control (TH > CS), dUCH knockdown (TH > dUCH.IR), and dUCH knockdown larvae treated with curcumin (TH > dUCH.IR + curcumin) using an anti-TH antibody with a lower (A–C) and higher magnification (A1–C1). DL1 clusters are enclosed with white boxes, and scale bars indicate 100 *μ*m (A–C) and 20 *μ*m (A1–C1). (d) Quantification of dopaminergic neurons in the DL1 cluster. The knockdown of dUCH caused a decrease in the number of dopaminergic neurons in the DL1 cluster, while dUCH knockdown larvae treated with curcumin did not show the degeneration of dopaminergic neurons (Tukey's test, ^∗∗∗^*P* < 0.001 and ^∗∗∗∗^*P* < 0.0001, *n* = 10). Error bars represent the standard deviation. TH > CS (*w/Y; +; TH-GAL4/+*) and TH > dUCH.IR (*w/Y; +; TH-GAL4/UAS-dUCH.IR*).

The above-described results on locomotor abilities suggested that curcumin might play a role in protecting dopaminergic neurons. Therefore, we analyzed the morphology of DA neuron clusters in larval and adult brains by immunostaining with an anti-tyrosine hydroxylase (anti-TH) antibody. In the larval stage, the brain lobes of dUCH knockdown larvae (*w/Y; +; TH-GAL4/UAS-dUCH.IR*) (Figure 5(b) B1) had fewer DA neurons in DL1 clusters (10 neurons) than those of control larvae (*w/Y; +; TH-GAL4/+*) (13 neurons) (Figure 5(a) A1). However, dUCH knockdown larvae treated with curcumin (Figure 5(c) C1) had more DA neurons in DL1 clusters (13 neurons) than curcumin-untreated knockdown larvae (10 neurons). The number of DL1 DA neurons was similar between curcumin-treated dUCH knockdown larvae and control larvae. These differences were significant (Tukey's test, *P* < 0.001, *n* = 10) (Figure 5(d)). Hence, treatments with curcumin may protect against DA neuron degeneration caused by the specific knockdown of dUCH in DA neurons. This may result in the recovery of crawling ability in knockdown larvae treated with curcumin.

**Figure 6:**
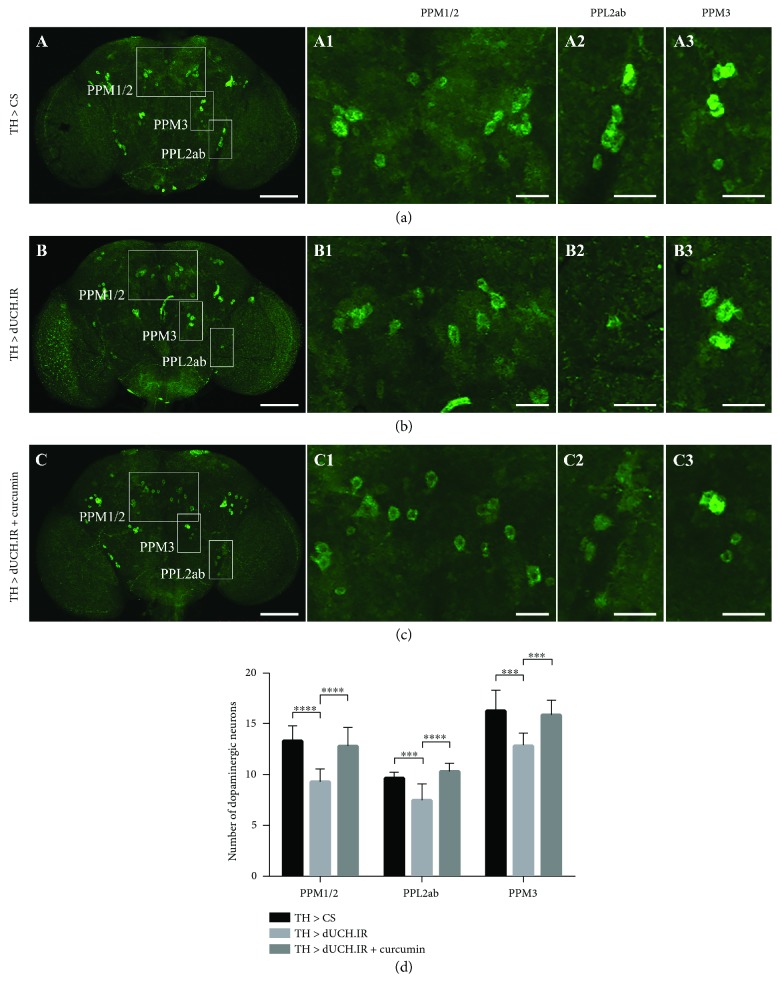
The curcumin treatment reduced the loss of dopaminergic neurons in adult brains caused by the knockdown of dUCH. (a–c) Images show dopaminergic neurons in adult brains stained with an anti-TH antibody. The indicated white boxes enclose the dopaminergic neuron clusters and are magnified on the right side of each panel (A1–C1, A2–C2, and A3–C3). Scale bars represent 100 *μ*m (A–C) and 25 *μ*m (A1–A3, B1–B3, and C1–C3). (d) The number of dopaminergic neurons in PPM1/2, PPL2ab, and PPM3 clusters from 15-day-old fly brains. The loss of dopaminergic neurons decreased in curcumin-treated flies with the knockdown of dUCH (Tukey's test, ^∗∗∗^*P* < 0.001 and ^∗∗∗∗^*P* < 0.0001, *n* = 10). Error bars represent the standard deviation of data. TH > CS (*w/Y; +; TH-GAL4/+*) and TH > dUCH.IR (*w/Y; +; TH-GAL4/UAS-dUCH.IR*).

Since we also found that the curcumin treatment resulted in the recovery of climbing ability in the adult stage, we evaluated the number of DA neurons in the adult brain. In this experiment, brains from 15-day-old flies were collected and examined. The results showed that the numbers of DA neurons in the PPM1/2, PPL2ab, and PPM3 clusters were lower in dUCH knockdown flies (9, 8, and 13 neurons, resp.) than in control flies (13, 10, and 16 neurons, resp.) (Figures 6(a) and 6(b)). The numbers of DA neurons in the PPM1/2, PPL2ab, and PPM3 clusters were higher in curcumin-treated knockdown flies (13, 10, and 16 neurons, resp.) (Figure 6(c)) than in knockdown flies not treated with curcumin (Figure 6(b)) and were similar to those in control flies (Figure 6(a)). These differences were significant (Tukey's test, *P* < 0.001, *n* = 10) (Figure 6(d)). Therefore, DA neuron degeneration was decreased by the curcumin treatment in not only the larval stage but also the adult stage.

### 3.5. The Degree of Dopamine Deficiencies in the dUCH Knockdown Fly Brain Was Reduced by the Curcumin Treatment

**Figure 7:**
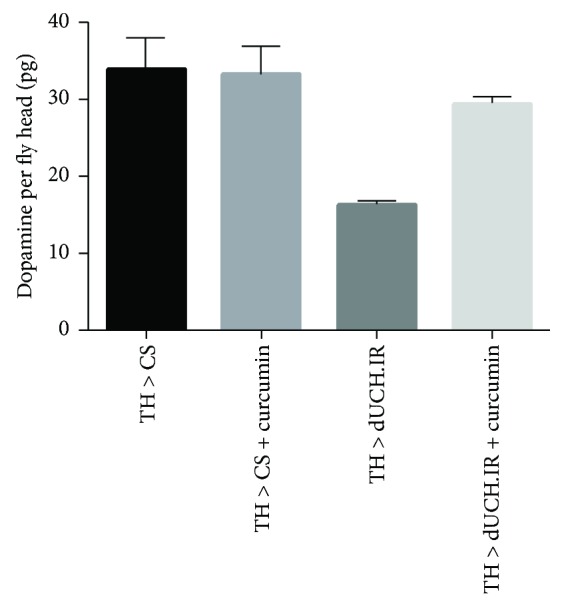
Quantification of dopamine in adult fly brains. Curcumin did not affect dopamine levels in control fly brains (TH > CS and TH > CS + curcumin). The knockdown of dUCH in dopaminergic neurons resulted in lower dopamine levels in knockdown fly brains (TH > dUCH.IR) than in control fly brains (TH > CS). Curcumin reduced the negative impact of the dUCH knockdown on dopamine levels. Curcumin-treated knockdown flies (TH > dUCH.IR + curcumin) showed only slightly lower dopamine levels than those of control flies (TH > CS) (population size *N* = 30 and biological replication *n* = 2; error bars indicate the standard deviation of data). TH > CS (*w/Y; +; TH-GAL4/+*) and TH > dUCH.IR (*w/Y; +; TH-GAL4/UAS-dUCH.IR*).

A decrease in the level of the neurotransmitter dopamine has been reported in PD patients and is a factor leading to clinical symptoms [40]. Therefore, we investigated the effects of a curcumin treatment on dopamine levels in adult brains. In this assay, we tested dopamine levels in the brains of 15-day-old flies. The results showed that dopamine levels were 52% lower in dUCH knockdown fly brains (*w/Y; +; TH-GAL4/UAS-dUCH.IR*) than in control fly brains (*w/Y; +; TH-GAL4/+*). However, dUCH knockdown flies treated with curcumin showed only a slight decrease in dopamine levels of 13% from those in control flies, while curcumin did not impact dopamine levels in control flies (Figure 7). This assay was repeated twice with similar results, and coefficients of variation were less than 17%. These results suggest that curcumin has the potential to protect DA neurons and reduce the degree of dopamine deficiencies.

## 4. Discussion

PD is a neurodegenerative disorder caused by environmental and genetic factors. However, the onset mechanisms of PD currently remain unclear, and this represents an obstacle to the development of effective treatments. Previous studies demonstrated that oxidative stress is one of the main causes of PD. The formation of Lewy bodies and oxidative stress have been found in the pars compacta of the substantia nigra of PD patients [41–44]. Therefore, antioxidants are considered to be a promising approach to decelerate the progression of PD. Curcumin is regarded as a powerful antioxidant [45]. Previous studies reported the neuroprotective properties of curcumin in *in vitro* and *in vivo* PD models induced by several different environmental factors such as neurotoxins and genetic factors including *α-synuclein*, *PINK1*, *DJ-1*, and *LRRK2* [22–24, 26–31].

In the present study, we established a *Drosophila* model with the knockdown of dUCH, which is a homolog of human UCH-L1. Setsuie and Wada previously reported a relationship between the abnormal expression, structure, and function of UCH-L1 and neurodegenerative diseases including PD [46]. UCH-L1 plays important roles in UPS. A dysfunction in UPS may lead to protein aggregation and oxidative stress [9, 12, 47]. This is consistent with the present results obtained from dUCH knockdown flies. We revealed that the knockdown of dUCH resulted in elevated ROS levels in larval eye imaginal discs and adult brains. Moreover, we found that ROS levels in eye imaginal discs and adult brains decreased significantly in dUCH knockdown flies fed curcumin. These results provided strong evidence to support the relationship between the knockdown of dUCH and oxidative stress.

*Drosophila* has a potential as a model for studying PD [32]. Fly models have been reported to show strong PD-like phenotypes characterized by locomotion impairments and dopaminergic neuron (DA neurons) degeneration as well as defects associated with mitochondrial dysfunction, oxidative stress, and protein aggregation [48]. In this model, we found that the DA neuron-specific knockdown of dUCH resulted in locomotive disabilities, DA neuron degeneration, and decreases in dopamine levels. These phenotypes are consistent with PD symptoms in humans, including locomotive defects and the loss of DA neurons [5–7], and PD-like phenotypes in other fly models [48]. The curcumin treatment ameliorated the PD-like phenotypes induced by the knockdown of dUCH. The DA neuron-specific knockdown of dUCH exerted long-term adverse effects on locomotor abilities in the larval and adult stages. However, the crawling ability of dUCH knockdown larvae including average speeds and motion paths strongly recovered after knockdown larvae had been treated with curcumin. Similarly, the curcumin treatment improved the climbing ability of dUCH knockdown flies. We also found that DA neuron degeneration was decreased by the curcumin treatment in the larval and adult stages. Furthermore, the extent of the depletion of dopamine was reduced after flies had been treated with curcumin. Therefore, we suggest that treatments with curcumin protect against the negative effects of dUCH knockdown on locomotor abilities, the number of DA neurons, and dopamine levels.

The neuroprotective properties of curcumin have been demonstrated in studies using many PD models induced by several different environmental and genetic factors. In addition to models developed by manipulations of the *α-synuclein*, *PINK1*, *DJ-1*, and *LRRK2* genes, curcumin also exerted positive effects on dUCH knockdown flies. Significant improvements were observed in PD-like phenotypes in dUCH knockdown flies, which were characterized by progressive locomotor defects accompanied by the degeneration of DA neurons. Since curcumin exerts neuroprotective effects on PD models induced by several different environmental factors (such as 6-OHDA, MPTP, rotenone, and paraquat) and genetic factors (including *α-synuclein*, *PINK1*, *DJ-1*, *LRRK2*, and *UCH-L1*), our results provide additional evidence to demonstrate that treatments with curcumin have a potential as an appropriate therapy for PD related to oxidative stress.

## Data Availability

The datasets generated during the current study are available from the corresponding author on reasonable request..
